# A parabolic model of drag coefficient for storm surge simulation in the South China Sea

**DOI:** 10.1038/srep15496

**Published:** 2015-10-26

**Authors:** Shiqiu Peng, Yineng Li

**Affiliations:** 1 State Key Laboratory of Tropical Oceanography, South China Sea Institute of Oceanology, Chinese Academy of Sciences, Guangzhou, China, 510301

## Abstract

Drag coefficient (*C*_*d*_) is an essential metric in the calculation of momentum exchange over the air-sea interface and thus has large impacts on the simulation or forecast of the upper ocean state associated with sea surface winds such as storm surges. Generally, *C*_*d*_ is a function of wind speed. However, the exact relationship between *C*_*d*_ and wind speed is still in dispute, and the widely-used formula that is a linear function of wind speed in an ocean model could lead to large bias at high wind speed. Here we establish a parabolic model of *C*_*d*_ based on storm surge observations and simulation in the South China Sea (SCS) through a number of tropical cyclone cases. Simulation of storm surges for independent Tropical cyclones (TCs) cases indicates that the new parabolic model of *C*_*d*_ outperforms traditional linear models.

Historically, a diversity of the relationship between *C*_*d*_ and wind speed has been employed in numerical models or analysis for the calculation of momentum exchange over the air-sea interface (Fig. 1). Theoretically, Drag coefficient (*C*_*d*_) is a function of sea surface roughness which is determined by a number of factors, including wind speed, wave, spume, flying spray, and atmospheric stability^1^,^2^:


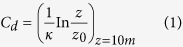


Here *κ*(=0.40) is the von Kármán constant and *z*_0_ is the sea surface roughness length for wind speed.

Practically and historically, however, *C*_*d*_ had commonly been set either as a constant^3^,^4^,^5^ or using an empirical formula that is a linear function of wind speed^6^,^7^,^8^,^9^,^10^,^11^ or leveling off at high wind speed^12^,^13^ (Fig. 1). Recently, more and more nonlinear formulas are proposed and applied in practice^14^,^15^,^16^. In real situation, extensive wave-breaking generated by high winds could result in a thin lay of white crest (including spume, flying spray, etc) which acts like a shroud shielding the fine scale wave roughness from the airflow and thus reduces the roughness of sea surface and *C*_*d*_. In the past decade, field measurements indicate that *C*_*d*_ reaches a maximum near 30–40 m s^−1^ and then decreases with increasing wind speed^17^,^18^,^19^,^20^,^21^,^22^. The results of Jarosz *et al.* (2007) and Sahlée *et al.* (2012) also show that when using most of presented empirical formulas *C*_*d*_ is underestimated under the intermediate wind speed (especially for those linearly-increasing formulas under intermediate wind speed) or overestimated under the very high wind speed (especially for those non-decreasing formulas), inevitably leading to biases in the wind stress calculation. Therefore, to reduce the biases, some nonlinear formulas are proposed and applied in practice in recent years^14^,^15^,^16^.

As shown by Jarosz *et al.* (2007), the variation of *C*_*d*_ with wind speed seems to be a parabolic function. Because a parabolic function generally produces larger (smaller) values of *C*_*d*_ than a linear function before (after) reaching its maximum, it may represent more accurately the variations of *C*_*d*_ under intermediate and very high wind speeds compared to a linear function. In addition, a parabolic function has only two parameters to be determined, and thus is easier to be estimated using 4-Dimentional Variational Data Assimilation (4DVAR) approach. Therefore, we hypothesize that *C*_*d*_ is a parabolic function of wind speed under intermediate and high wind speeds. Based on an ensemble of typhoon cases, here we establish an “optimal” model of *C*_*d*_ in the frame of parabolic shape for the South China Sea (SCS) through assimilating the observed water level into a storm surge model using 4DVAR technique.

## Results

Based on the maximum value of *C*_*d*_ around (32–33 m/s) in the field measurements of Powell *et al.* (2003) and Jarosz *et al.* (2007), we propose a parabolic form for the relationship between *C*_*d*_ and wind speed:


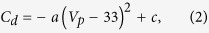


where *V*_*p*_ is the sea surface wind speed at 10 m height, and *a* and *c* are the two parameters to be determined. The initial values of *a* and *c* are set to be (*a*_*0*_, *c*_*0*_) = (2.0 × 10^−6^, 2.34 × 10^−3^). Over the continental shelf and the coastal regions, the forced response consists of a strong barotropic component that is not geostrophically balanced and a much weaker baroclinic component, especially during the passage of a TC. Thus, we can use a storm surge model and coastal water level observations to determine the parameters (*a*, *c*) through 4DVAR technique.

**Figure 1:**
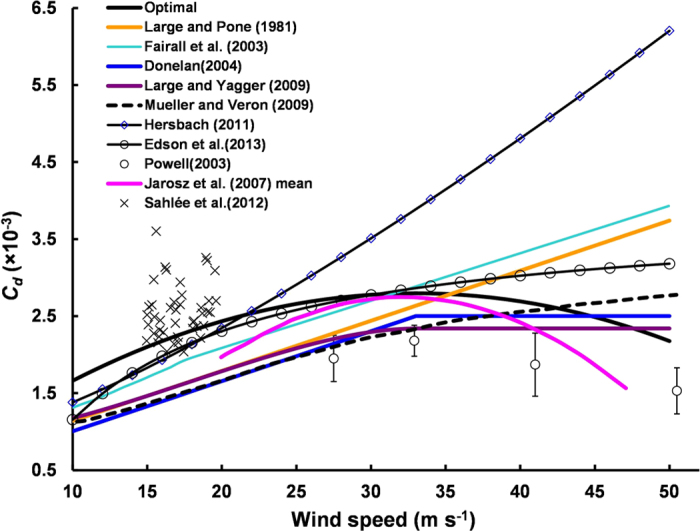
Wind stress drag coefficient (*C*_*d*_) as a function of wind speed (Unit: m s^−1^) from the parabolic model and other models. Parabolic model (black), Large and Pond (1981, ref. 8; orange), Fairall *et al.* (2003, ref. 11; sky-blue), Donelan *et al.* (2004, ref. 12; blue), Large and Yagger (2009, ref. 13; purple), Mueller and Veron (2009, ref. 14; dashed), Hersbach (2011, ref. 15; rhombus and black line), Edson *et al.* (2013, ref. 16; circle and black line), Powell *et al.* (2003, ref. 17; circle), Jarosz *et al.* (2007, ref. 19; peach) and Sahlée *et al.* (2012, ref. 38; cross).

**Figure 2:**
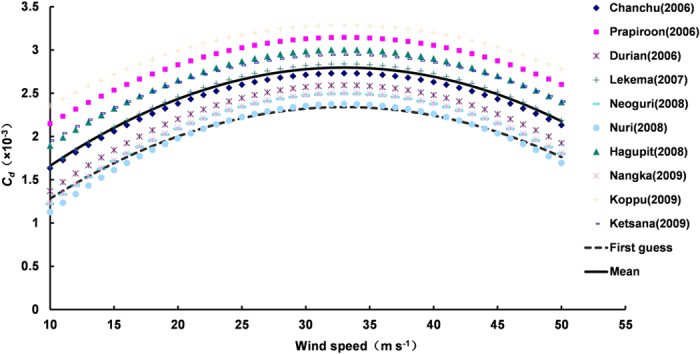
The parabolic model of *C*_*d*_ as a function of 10 m wind speed (Unit: m s^−1^) optimized for each of TC Cases I. The black dashed and solid lines represent the first guess and the mean, respectively.

The 4DVAR technique has been widely employed to optimize both the model initial conditions and physical parameters, which involves forward and backward (adjoint) model integration when minimizing the misfit between model output and observations^23^,^24^,^25^,^26^,^27^,^28^,^29^,^30^. To determine and validate Equation (2) at high wind speeds, we select all the relatively strong typhoon cases that originated in or passed through the SCS regions with storm surges induced at least 0.2 m in the coast during 2006–2011, counting a number of 18 (see Table S1 in Supplementary Information Online); Ten of the selected cases (denoted as Cases I) are used to determine the values of *a* and *c* in Equation (2), the rest (denoted as Cases II) are used for validation. For each of Cases I, a parabolic function of *C*_*d*_ with respect to wind speed is obtained by optimizing *a* and *c* to minimize the distance between the observed and modeled storm surges through 4DVAR (Fig. 2 and Table 1). The mean of the ensemble of the parabolic functions is then adopted as the parabolic model of *C*_*d*_ with respect to the wind speed for the SCS region (Fig. 2 and Table 1). The new parabolic model obtained here has a trend similar to those of Powell *et al.* (2003) and Jarosz *et al.* (2007) based on field measurements with slightly large magnitude in the whole band of medium to high wind speed (Fig. 1), and gives larger values of *C*_*d*_ than most of the other models under intermediate wind speed. The storm surge simulations for Cases I using the new model of *C*_*d*_ gain significant improvements with smaller maximum surge biases and Root Mean Square Errors (RMSE) compared to those using other models of *C*_*d*_ (see Supplementary Information online Table S2–3).

The new model of *C*_*d*_ is then employed in the storm surge model for the 8 typhoons of Cases II to validate its effect on improving storm surge simulations in the SCS. Compared to other models of *C*_*d*_, the parabolic model of *C*_*d*_ statistically produces better storm surge simulations with smaller maximum surge biases and RMSE (see Table 2).

It should be aware that the values of parameters *a* and *c* in the parabolic model are determined based on the storm surge observations associated with relatively strong typhoons in the SCS region. For other regions, the parabolic relationship between *C*_*d*_ and wind speed can be still applicable but the values of parameters *a* and *c* should be determined based on observations in those regions using the same method. Therefore, our results reported here provide not only a new parabolic model of *C*_*d*_ applicable for the SCS region, but also a practical way to establish a parabolic model of *C*_*d*_ for other coastal regions.

## Methods

**Figure 3:**
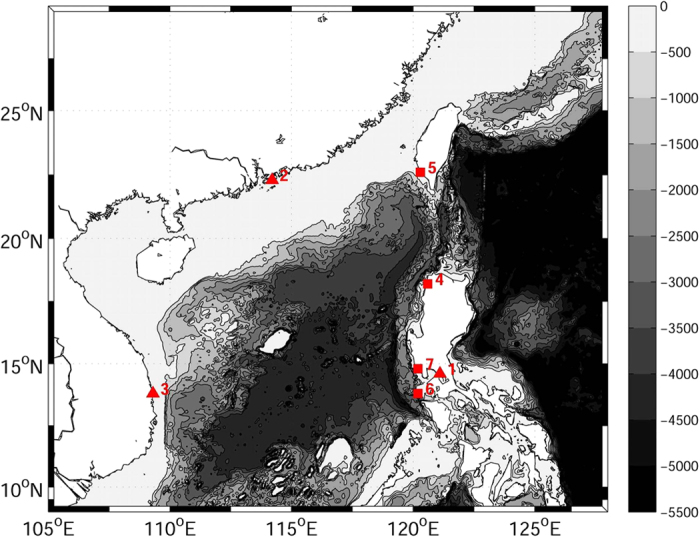
Map of the bathymetry (Unit: m) of the model domain with locations of water level stations. Triangle indicates the station used for *C*_*d*_ optimization, while square indicates the station used for validation. The model domain covers most of the South China Sea (SCS) and part of the northern West Pacific. (The figure is plotted by MATLAB software with M_Map package).

The surge observations and the reconstructed wind data are used for adjusting initial conditions (ICs) and parameters (*a*, *c*) to obtain the ensemble of “optimal” parameters. The sea level data are from the research quality data set of Joint Archive for Sea level (JASL) which is provided by the University of Hawaii Sea-level Center (UHSLC)^31^. The JASL receives hourly data from regional and national sea level networks. The data were inspected and obvious errors such as data spikes and time shifts were corrected. Gaps less than 25 hours were interpolated. In this study, we focus on the storm surges caused by Tropical cyclones (TCs) in the SCS. Thus, 7 stations are selected for the optimization of *C*_*d*_ and validation (Fig. 3 and Table S4 in Supplementary Information Online). Three of the selected stations (number 1–3) are used for optimizing *C*_*d*_ through 4DVAR and the rest (number 4–7) are used for the validation. The tidal information in the observations is removed by subtracting tidal component (obtained by the harmonic analysis) from the original sea level. After that, there is still high frequency information in the data, thus a filtration with 3-hour period is performed before the optimization of *C*_*d*_ and further analysis.

As the wind biases are large for satellite analysis data around the center of TC and for the empirical TC wind model far from TC center, we reconstruct a sea surface wind data by combining the satellite analysis wind and the empirical model wind. In this study, Holland model is used for the calculation of empirical TC wind^32^. The tangential wind speed from the empirical Holland model, which is based on the balance between the pressure gradient and centrifugal forces, can be expressed as


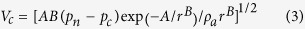



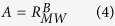


where *A* and *B* are the scaling parameters, *p*_*n*_ and *p*_*c*_ are the ambient and central pressure of the storm, respectively, *ρ*_*a*_ is the air density, *r* is the distance from the storm center, and *R*_*MW*_ is the radius of the maximum wind (RMW, the distance between the center of a cyclone and its band of the strongest wind). Here, the best track data from Joint Typhoon Warning Center (JTWC) are used^33^. Empirically, *B* lies between 1 and 2.5. In this study, *B* is set to be 1.7 which is the median of the range. The satellite analysis wind is from the 6-hourly Cross-Calibrated Multi-Platform (CCMP) wind data with a spatial resolution of 0.25°. The CCMP data set combines data derived from SSM/I, AMSRE, TRMM TMI, QuikSCAT, and other missions using a variational analysis method (VAM) to produce a consistent climatological record of ocean surface vector winds at 25-km resolution^34^. To combine the two data sets we introduce a weight coefficient,


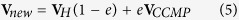


where **V**_*H*_ is the wind data from Holland model, **V**_*CCMP*_ is the wind data from CCMP. The weight coefficient *e* is defined as *e* = *C*^4^/(1 + *C*^4^), and *C* = *r*/(*nR*_*MW*_) is a coefficient measuring the area affected by a TC. *r* is the distance between the center of a cyclone and the calculation point. Empirically, parameter *n* is set to 9 or 10 (compared with the maximum wind speed of JTWC, n is set to be 9 in this study). **V**_*new*_ is assumed to be the realistic wind and used for the adjustment of *C*_*d*_.

The 4DVAR system used for the adjustment of *C*_*d*_ is based on the 2002 version of Princeton Ocean Model (POM2k)^35^ as well as its tangent linear and adjoint models^29^. The POM2k is a three dimensional, fully nonlinear, primitive equation ocean model, and the 2.5-order turbulence closure scheme of Mellor and Yamada (1982) to calculate turbulence viscosity and diffusivity^36^. Due to the limited space here, we refer the readers to Peng and Xie (2006) for details on the linearization of the vertical turbulence scheme as well as other issues related to the tangent linear and adjoint models of POM2k. In this study, ICs and parameters *a* and *c* of *C*_*d*_ are chosen as the control variables in the adjoint model (as proposed by Li *et al.* (2013)). The cost function with ICs and parameters *a* and *c* being the control variables is defined as a misfit between the model and the observations, i.e.


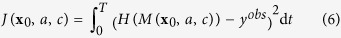


where **x**_**0**_ represents the ICs, *M* nonlinear ocean model*, H* the observation operator, *y*^*obs*^ the observation variables, and *T* the assimilation time window. The cost function is calculated when observations are available and the absolute value of surge is over 0.1 m. In addition, the wind speed data used for the estimation range from 10 m s^−1^ to 70 m s^−1^ for the selected cases. In order to find the optimal values for *a* and *c*, the minimization of cost function is performed. It is achieved by obtaining its gradient with respect to the control variables X_0_, *a* and *c* by integrating the adjoint model of POM2k backward in time. The limited memory Broyden-Fletcher-Goldfarb -Shanno (BFGS) quasi-Newton minimization algorithm^37^ is employed to obtain the optimal control variables. The optimization process is illustrated by the flowchart shown in Fig. S1 of the Supplementary Information online.

## Additional Information

**How to cite this article**: Peng, S. and Li, Y. A parabolic model of drag coefficient for storm surge simulation in the South China Sea. *Sci. Rep.* **5**, 15496; doi: 10.1038/srep15496 (2015).

## Supplementary Material

Supplementary Information

## Figures and Tables

**Table 1:** The “optimal” values of parameters (*a*, *c*) and their mean after data assimilation.

No.	Typhoon	*a*	*c*
1	Chanchu	0.00212	2.787
2	Prapiroon	0.00188	3.146
3	Durian	0.00231	2.593
4	Lekima	0.00226	2.839
5	Neoguri	0.00241	2.495
6	Nuri	0.00236	2.376
7	Hagupit	0.00210	3.003
8	Nangka	0.00240	2.503
9	Koppu	0.00176	3.287
10	Ketsana	0.00188	2.945
	Mean	0.00215	2.797

**Table 2:** Biases and Standard Deviation (SD) of maximum storm surge (Units: m) and Root-Mean-Squared-Errors (RMSE) of storm surge (Units: m) (in parenthesis) simulated by different *C*_*d*_ models for TC Cases II.

No.	Typhoon	Large& Pond(1981)	Donelan(2004)	Large &Yagger(2009)	Fairall *et al*.(2003)	Mueller andVeron(2009)	Hersbach(2011)	Edson*et al*.(2013)	Firstguess	Optimal
1	Conson	0.129(0.096)	0.110(0.097)	0.125(0.094)	0.161(0.092)	0.121(0.095)	0.237(0.088)	0.208(0.105)	0.152(0.099)	0.115(0.095)
2	Meranti	−0.07(0.076)	−0.075(0.08)	−0.071(0.074)	−0.063(0.067)	−0.072(0.08)	−0.043(0.064)	−0.064(0.072)	−0.067(0.071)	−0.053(0.065)
3	Megi	−0.153(0.138)	−0.191(0.151)	−0.201(0.141)	−0.138(0.129)	−0.19(0.147)	−0.036(0.148)	−0.147(0.128)	−0.207(0.133)	−0.171(0.112)
4	Haima	−0.222(0.141)	−0.222(0.141)	−0.212(0.179)	−0.204(0.173)	−0.228(0.149)	−0.203(0.163)	−0.221(0.138)	−0.209(0.174)	−0.193(0.139)
5	Nock-ten	−0.07(0.055)	−0.087(0.065)	−0.063(0.05)	−0.044(0.043)	−0.1(0.077)	−0.04(0.043)	−0.075(0.059)	−0.056(0.049)	−0.012(0.04)
6	Nanmadol	−0.097(0.112)	−0.116(0.121)	−0.109(0.117)	−0.079(0.108)	−0.109(0.129)	−0.029(0.099)	−0.063(0.122)	−0.101(0.12)	−0.064(0.11)
7	Nesat	−0.233(0.167)	−0.259(0.168)	−0.231(0.169)	−0.192(0.166)	−0.254(0.162)	−0.166(0.168)	−0.186(0.158)	−0.2(0.164)	−0.13(0.168)
8	Nalgae	−0.017(0.136)	−0.052(0.14)	−0.016(0.137)	0.037(0.14)	−0.034(0.137)	0.092(0.144)	0.082(0.14)	0.034(0.134)	0.072(0.142)
	SD(Mean RMSE)	0.143(0.115)	0.155(0.120)	0.148(0.120)	0.131(0.115)	0.156(0.122)	0.132(0.115)	0.145(0.115)	0.145(0.118)	0.117(0.109)

Shown in the bottom are the Standard Deviation (SD) of maximum storm surge and the mean RMSE (in parenthesis).
