# Supplementary material for: A parabolic model of drag coefficient for storm surge simulation in the South China Sea
Source: Sci Rep. 2015 Oct 26;5:15496. doi: 10.1038/srep15496 (PMC4620453; doi:10.1038/srep15496)
Supplement: Supplementary Information [file srep15496-s1.doc]

**A parabolic model of drag coefficient for storm surge simulation in the South China Sea**

**(Supplementary Information)**

Shiqiu Peng^1^ and Yineng Li^1^

1. State Key Laboratory of Tropical Oceanography, South China Sea Institute of Oceanology, Chinese Academy of Sciences, Guangzhou, China, 510301

To be submitted to Scientific Reports

April 30, 2015

Table S1 The selected TCs which can induce storm surge of over 0.2 m in the SCS from 2006 to 2011.

| NO. | Years | Name of TC | Min SLP  (hPa) | Max U_10_  (m s^-1^) | Simulating date time  (UTC) | Spin-up (forecast) time  (hour) | Maximum storm surge (m) | Adjusted *C_d_* or Independent test |
| --- | --- | --- | --- | --- | --- | --- | --- | --- |
| 1 | 2006 | Chanchu | 916 | 64 | 1012 0000 May to 1500 0000 May | 24 (84) | 0.415 | Adjusted *C_d_* |
| 2 | 2006 | Prapiroon | 972 | 35 | 3106 0000 July to 0406 0000 August | 24 (72) | 0.497 | Adjusted *C_d_* |
| 3 | 2006 | Durian | 904 | 69 | 2806 0000 November to 0506 0000 December | 48 (120) | 0.272 | Adjusted *C_d_* |
| 4 | 2007 | Likema | 970 | 36 | 3006 0000 September to 0406 October | 24 (72) | 0.228 | Adjusted *C_d_* |
| 5 | 2008 | Neoguri | 948 | 51 | 1400 0000 April to 1918 0000 April | 24 (114) | 0.318 | Adjusted *C_d_* |
| 6 | 2008 | Nuri | 948 | 51 | 1818 0000 August to 2218 0000 August | 24 (72) | 0.714 | Adjusted *C_d_* |
| 7 | 2008 | Hagupit | 929 | 64 | 2000 0000 September to 2500 00000 September | 24 (96) | 1.116 | Adjusted *C_d_* |
| 8 | 2009 | Nangka | 993 | 23 | 2318 0000 June to 2618 June | 24 (48) | 0.23 | Adjusted *C_d_* |
| 9 | 2009 | Koppu | 967 | 39 | 1206 0000 September to 1506 0000 September | 24 (48) | 0.488 | Adjusted *C_d_* |
| 10 | 2009 | Ketsana | 955 | 46 | 2518 0000 September to 2918 0000 September | 24 (72) | 0.41 | Adjusted *C_d_* |
| 11 | 2010 | Conson | 963 | 41 | 1112 0000 July to 1718 0000 July | 24 (120) | 0.223 | Independent test |
| 12 | 2010 | Meranti | 974 | 33 | 0718 0000 September to 1012 0000 September | 18 (48) | 0.208 | Independent test |
| 13 | 2010 | MEGI | 903 | 82 | 1612 0000 October to 2306 0000 October | 24 (138) | 0.604 | Independent test |
| 14 | 2011 | Haima | 993 | 18 | 2112 0000 June to 2412 0000 June | 24 (48) | 0.275 | Independent test |
| 15 | 2011 | Nock-ten | 974 | 33 | 2512 0000 July to 3012 0000 July | 24 (96) | 0.22 | Independent test |
| 16 | 2011 | Nanmadol | 918 | 72 | 2312 0000 August to 2918 August | 24 (126) | 0.268 | Independent test |
| 17 | 2011 | Nesat | 937 | 59 | 2400 0000 September to 3012 0000 September | 24 (132) | 0.545 | Independent test |
| 18 | 2011 | Nalgae | 926 | 67 | 2800 0000 September to 0506 0000 September | 24 (150) | 0.478 | Independent test |

Table S2 Biases and Standard Deviation (SD) of maximum storm surge (Units: m) simulated by different *C_d_* models for TC Cases I.

| **No.** | **Typhoon** | **Large & Pond (1981)** | **Donelan (2004)** | **Large & Yagger (2009)** | **Fairall et al. (2003)** | **Mueller et at. (2009)** | **Hersbach et al. (2011)** | **Edson et al. (2013)** | **First guess** | **Optimal** |
| --- | --- | --- | --- | --- | --- | --- | --- | --- | --- | --- |
| 1 | Chanchu | -0.148 | -0.164 | -0.151 | -0.124 | -0.154 | -0.109 | -0.11 | -0.126 | -0.085 |
| 2 | Prapiroon | -0.285 | -0.304 | -0.286 | -0.256 | -0.298 | -0.23 | -0.241 | -0.262 | -0.209 |
| 3 | Durian | 0.013 | 0.012 | 0.013 | 0.015 | 0.012 | 0.015 | 0.014 | 0.014 | 0.025 |
| 4 | Lekima | -0.026 | -0.035 | -0.025 | -0.011 | -0.033 | -0.006 | -0.01 | -0.013 | 0.014 |
| 5 | Neoguri | -0.087 | -0.108 | -0.093 | -0.057 | -0.104 | -0.022 | -0.056 | -0.069 | -0.015 |
| 6 | Nuri | -0.405 | -0.418 | -0.414 | -0.387 | -0.422 | -0.341 | -0.373 | -0.403 | -0.362 |
| 7 | Hagupit | 0.251 | -0.005 | -0.136 | 0.332 | 0.035 | 0.859 | 0.206 | -0.135 | 0.027 |
| 8 | Nangka | -0.157 | -0.165 | -0.154 | -0.144 | -0.167 | -0.14 | -0.154 | -0.148 | -0.126 |
| 9 | Koppu | -0.083 | -0.133 | -0.16 | -0.049 | -0.134 | 0.083 | -0.066 | -0.133 | -0.072 |
| 10 | Ketsana | -0.165 | -0.182 | -0.165 | -0.138 | -0.171 | -0.124 | -0.121 | -0.138 | -0.093 |
|  | SD | 0.199 | 0.196 | 0.195 | 0.197 | 0.195 | 0.310 | 0.173 | 0.181 | 0.146 |

Table S3 Root-Mean-Squared-Errors (RMSE) of storm surge (Units: m) simulated by different *C_d_* models for TC Cases I.

| **No.** | **Typhoon** | **Large & Pond (1981)** | **Donelan (2004)** | **Large & Yagger (2009)** | **Fairall et al. (2003)** | **Mueller et at. (2009)** | **Hersbach et al. (2011)** | **Edson et al. (2013)** | **First guess** | **Optimal** |
| --- | --- | --- | --- | --- | --- | --- | --- | --- | --- | --- |
| 1 | Chanchu | 0.111 | 0.117 | 0.111 | 0.103 | 0.114 | 0.099 | 0.101 | 0.104 | 0.096 |
| 2 | Prapiroon | 0.189 | 0.195 | 0.188 | 0.179 | 0.194 | 0.176 | 0.18 | 0.181 | 0.165 |
| 3 | Durian | 0.126 | 0.127 | 0.126 | 0.124 | 0.128 | 0.124 | 0.126 | 0.125 | 0.125 |
| 4 | Lekima | 0.13 | 0.131 | 0.13 | 0.13 | 0.13 | 0.13 | 0.128 | 0.13 | 0.131 |
| 5 | Neoguri | 0.135 | 0.139 | 0.132 | 0.129 | 0.14 | 0.13 | 0.133 | 0.13 | 0.122 |
| 6 | Nuri | 0.244 | 0.254 | 0.247 | 0.236 | 0.252 | 0.223 | 0.223 | 0.243 | 0.217 |
| 7 | Hagupit | 0.159 | 0.158 | 0.212 | 0.153 | 0.16 | 0.217 | 0.14 | 0.147 | 0.121 |
| 8 | Nangka | 0.132 | 0.133 | 0.13 | 0.13 | 0.136 | 0.129 | 0.133 | 0.131 | 0.127 |
| 9 | Koppu | 0.112 | 0.118 | 0.112 | 0.104 | 0.117 | 0.1 | 0.102 | 0.106 | 0.092 |
| 10 | Ketsana | 0.181 | 0.182 | 0.18 | 0.178 | 0.183 | 0.178 | 0.181 | 0.179 | 0.177 |
|  | Mean | 0.152 | 0.155 | 0.157 | 0.147 | 0.155 | 0.151 | 0.145 | 0.148 | 0.137 |

Table S4 The informations of the sea level stations.

| Number of stations | Name of stations | Longitude | Latitude | Frequency | Starting date | Ending date | For optimization (OPT) or Validation (VAL) |
| --- | --- | --- | --- | --- | --- | --- | --- |
| 1 | Manila | 121.080 | 14.640 | Hourly | Match 31, 2006 | April 3, 2012 | OPT |
| 2 | Quarry Bay | 114.220 | 22.300 | Hourly | January 1, 1986 | December 31, 2012 | OPT |
| 3 | Qui Nhon | 109.254 | 13.775 | Hourly | October 19, 2007 | April 3, 2012 | OPT |
| 4 | Currmao | 120.97 | 18.17 | Hourly | October 9, 2009 | April 15, 2012 | VAL |
| 5 | Kaohsiung | 120.283 | 22.617 | Hourly | January 1, 1985 | August 31, 2013 | VAL |
| 6 | Lubang | 120.210 | 13.820 | Hourly | June 11, 2010 | April 3, 2012 | VAL |
| 7 | Subic Bay | 120.283 | 14.817 | Hourly | February 27, 2007 | April 3, 2012 | VAL |

**Figure Legends**


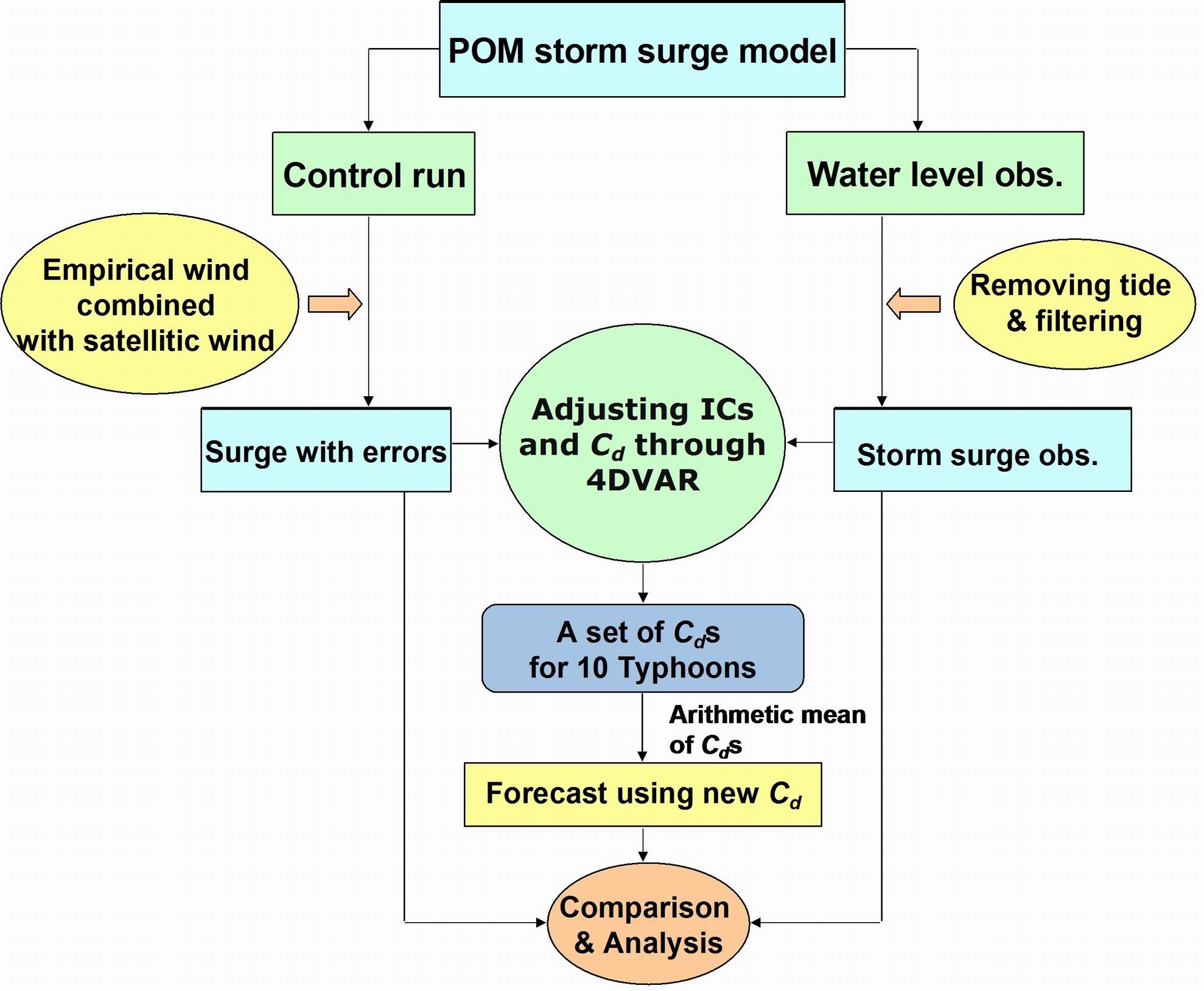


**Figure S1 Flowchart for the adjustment of *C_d_*.**
